# Dynamic Knee Alignment and Collateral Knee Laxity and Its Variations in Normal Humans

**DOI:** 10.3389/fsurg.2015.00062

**Published:** 2015-11-25

**Authors:** Kamal Deep, Frederic Picard, Jon V. Clarke

**Affiliations:** ^1^Golden Jubilee National Hospital, Glasgow, UK; ^2^University of Strathclyde, Glasgow, UK

**Keywords:** alignment, knee joint, ligament laxity, normal human, computer navigation, measurement

## Abstract

Alignment of normal, arthritic, and replaced human knees is a much debated subject as is the collateral ligamentous laxity. Traditional quantitative values have been challenged. Methods used to measure these are also not without flaws. Authors review the recent literature and a novel method of measurement of these values has been included. This method includes use of computer navigation technique in clinic setting for assessment of the normal or affected knee before the surgery. Computer navigation has been known for achievement of alignment accuracy during knee surgery. Now its use in clinic setting has added to the inventory of measurement methods. Authors dispel the common myth of straight mechanical axis in normal knees and also look at quantification of amount of collateral knee laxity. Based on the scientific studies, it has been shown that the mean alignment is in varus in normal knees. It changes from lying non-weight-bearing position to standing weight-bearing position in both coronal and the sagittal planes. It also varies with gender and race. The collateral laxity is also different for males and females. Further studies are needed to define the ideal alignment and collateral laxity which the surgeon should aim for individual knees.

## Introduction

**Figure 1:**
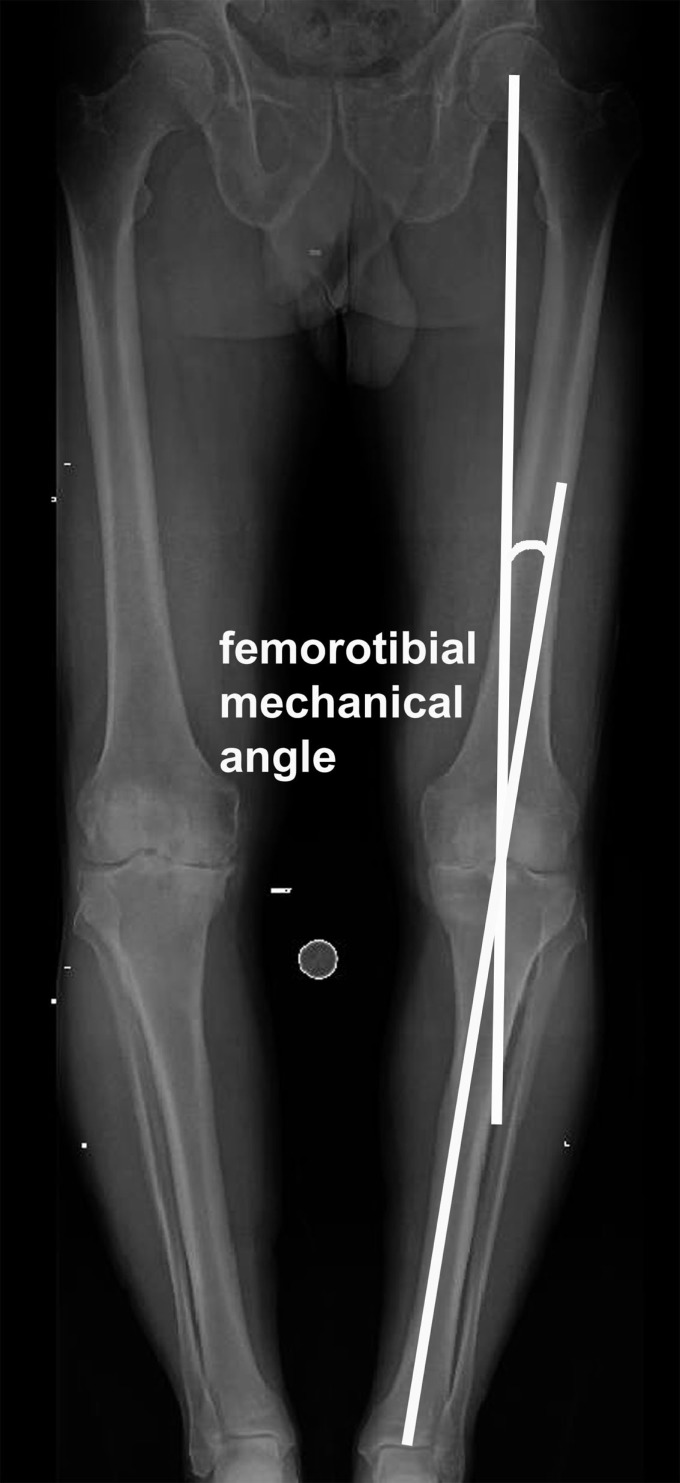
**The method of alignment measurement on plain radiographs**.

The shape and alignment of leg in normal humans has eluded surgeons for a long time. If it is straight, it is called neutral; if the ankle is going toward inside, it is called varus; and if the ankle is going toward outside, it is known as valgus alignment of the leg. The alignment is defined by joining the center of the hip, knee, and ankle joints, also known as mechanical axis of the leg (Figure 1). For calculation purpose, it is also known as femorotibial mechanical angle (FTMA). Traditionally the goal of total knee replacement arthroplasty (TKA) surgery has been to create a straight mechanical axis of the leg. The achievement of proper alignment in the coronal plane is important in maximizing the long-term success of this procedure. Many studies have implicated implanted component malalignment in the failure of TKA, wear, and loosening (1–3). While traditionally surgeons used fixed angle cuts with conventional surgical tools, this only achieved desired alignment in approximately two-thirds of patients. It is now known that the alignment may differ in different individuals. It is traditionally measured using radiographs that are subject to inaccuracies especially if leg is not positioned correctly or has arthritis and deformity. Recently computer-assisted navigation systems and patient-specific cutting blocks have been used to help orthopedic surgeons achieve individual patient-specific neutral alignment in knee replacement surgery (4–7). However, international arthroplasty registries in the United Kingdom, Canada, and New Zealand have shown that upto 20% of patients with TKA may be dissatisfied (8–10).

Anatomic variations in knee alignment exist not only between different ethnic races but also between males and females (11). Alignment of normal limbs in the coronal plane measured with computed tomography (CT) scanogram at the hip, knee, and ankle has shown that 98% of normal limbs do not have a neutral mechanical axis and 76% of normal limbs have a deviation of >3° from neutral (12). Therefore, the surgical correction of the arthritic knee to establish a straight mechanical axis does not represent a correction to “normal” alignment (12–14). It has been recently stated that non-weight-bearing alignment in lying position is different from weight-bearing standing position (15). This point is not very well understood by surgeons because of lack of studies and proper measurement methods in both postures.

Recently, computer navigation has been employed to evaluate the same on arthritic patients on outpatient basis (16). Even with most modern techniques like computer navigation, personalized cutting jigs, and implants, surgeons try to achieve patient-specific neutral, as opposed to patient-specific “normal” alignment for individual patients. This neutral alignment is targeted in non-weight-bearing supine position.

The balancing of the collateral (medial and lateral) ligaments is another aspect of knee surgery that is thought to be very important to achieve good results. Unfortunately, side to side (collateral) laxity of the ligaments in normal individuals is not understood either. The surgeons traditionally teach achieving around 2° of laxity on either side.

The understanding of “normal” knee alignment and collateral laxity in the knee joint specific to the individual person is necessary to further improve outcomes and satisfaction after total knee arthroplasty. One should also be careful about the limitations of the measurement methods used to assess these parameters.

## Methods of Measurement

A confusion is present in the existing literature due to different measurement methods and their accuracies. A lot of earlier literature is based on plain radiographs in non-weight-bearing standing position with short knee radiographs. More recent studies have included long-leg radiographs that include hip, knee, and ankle centers.

The routine standard measurement of knee alignment relies on clinical evaluation in conjunction with radiographs that center on the knee joint. However, human assessment of angles is known to be poor (17, 18) and the accuracy of alignment estimates under these circumstances may be variable (±5) (19). The use of knee radiographs has been found to be an inaccurate measure of mechanical lower limb alignment (20). Full-length hip-knee-ankle radiographs are susceptible to limb positioning errors with apparent variations in alignment produced as a result of knee flexion or rotation (21). CT imaging can overcome these positional artifacts by providing a 3D evaluation of lower limb anatomy but is unable to provide weight-bearing information. Further drawbacks of both the radiographs as well as CT scan modalities include exposure to ionizing radiation. Magnetic resonance (MR) scans have also been used to evaluate the knee anatomy. While long-leg radiographs can be taken in weight-bearing standing position, the CT and MR scans are done in non-weight-bearing supine position. Hence, the measurements on radiographs and CT or MR scans may not be comparable. While supine position compares well with position during surgery, it is not functional position that patient adopts in standing, walking, or running.

**Figure 2:**
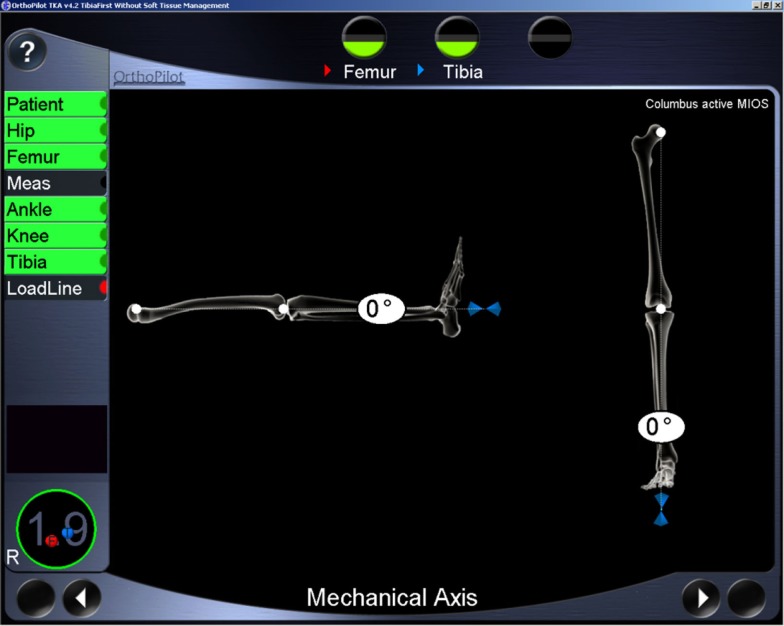
**The computer screen representation of leg alignment in coronal and sagittal planes**.

Computer navigation using infrared tracking has been introduced intra-operatively to provide surgeons with quantitative measurement tools that permit real-time assessment of lower limb kinematics (22–24). Now it is available to measure on outpatient basis as well which uses a similar infrared-based computer navigation system for measurement of FTMA that has been developed and validated (16). Inter and intra observer registration measurements using the extracutaneous straps have been validated using this method that uses a specialized fibro elastic strap attachment (16). The authors found the errors of up to 1°(16). Another study has used the same method for standardizing measurement of coronal laxity measurements (25). This method of measurement has a number of potential advantages over other measurement systems. The immediate generation of real-time on-screen coronal and sagittal FTMA angles (Figure 2) enables dynamic measurements of alignment to be made on weight bearing with immediate visualization of angular displacement. This is usable in outpatient clinic setting with no ionizing radiation. Use of pre- and intraoperative computer navigation may help the surgeon set better goals of alignment and kinematics for individual patients and achieve desired results intra-operatively in those patients. Thus, it can be a useful screening tool on outpatient basis. Also, the method can be used to evaluate and compare outcomes after total knee arthroplasty.

## Alignment and Its Variations

Authors have conducted studies to evaluate alignment in normal population using computerized measurement tool (26). We also looked at its dynamic nature and variations with posture, side, race, and gender. A prospective multicenter study on healthy volunteers aged between 19 and 35 was done. This included 267 knees of which 155 were from male and 112 from female subjects. A validated method of measurement using computerized infrared rays-based computer navigation system (Orthopilot, B Braun, Tutlingen) was used for measurement of the FTMA (16). The centers of the hip, knee, and ankle were acquired and registered into the computer. The computer produced a mechanical axis by joining these.

Surprisingly, the mean supine non-weight-bearing alignment was not straight, FTMA was not neutral but a varus of 1.2°(SD = 4°) (26).

Eckhofff and coauthors found in their study based on supine non-weight-bearing CT scan of 180 healthy legs, a mean deviation of FTMA of 2.7°(SD = 2.6) from the neutral (12). Bellemans and coauthors found in their study based on standing radiographs in 250 asymptomatic adults, mean FTMA was 1.3° varus (SD = 2.34) (13). The absolute values in different studies may be little different which may be explained by genetic constitution of the study populations, postural variation, or potential errors due to measurement methods used in the studies. The results of these studies indicate wide variation in FTMA even among “normal” individuals with functionally normal knees. Therefore, the surgical correction of the arthritic knee to establish a straight mechanical axis does not represent a correction to “normal” alignment (12–14).

Change in coronal alignment with standing has been published comparing supine computer navigation and standing long-leg radiographs in a study in the arthritic and replaced knees (27). Our study found this also happens in normal knees (26). The supine FTMA changed by a mean of varus 2.2°(SD = 3.6°) in bipedal weight-bearing stance and a mean varus of 3.4°(SD = 3.8°) in monopedal weight-bearing stance. Both these weight-bearing alignments changed significantly from non-weight-bearing supine alignment (*p*–value <0.001). The supine varus knees, neutral knees and those up to 2.5° valgus tended to go in varus direction on standing but more valgus tended to go in further valgus on standing (26).

Similar results are also noted in a study by Clarke et al. who studies 30 asymptomatic and 31 arthritic and replaced knees post TKR using same method of measurement (28). They found that alignment in both planes changed significantly from supine to standing for all three groups. For the coronal plane, the supine and standing measurements [in degrees, mean (SD)] were 0.1 (2.5) and −1.1 (3.7) in the asymptomatic group, −2.5 (5.7) and −3.6 (6) in the OA group and −0.7 (1.4), and −2.5 (2) in the TKA group. For the sagittal plane, the numbers were −1.7 (3.3) and −5.5 (4.9); 7.7 (7.1) and 1.8 (7.7); and 6.8 (5.1) and 1.4 (7.6), respectively (28). They also noted that change was most frequently toward relative varus and extension as we found in our study. They noted that the trend of relative varus and extension in standing stance was similar in overall magnitude and direction between the asymptomatic, arthritic, and the replaced groups. The authors concluded that the consistent kinematic pattern for different knee types suggests that soft tissue restraints rather than underlying joint deformity may be more influential in control of alignment from lying to standing (28). In the normal knee study, the sagittal plane the knee extension increased significantly by 5.6°(SD = 6.8°) in bipedal stance (*p*-value <0.001) and by 5.5°(SD = 7.7°) in monopedal stance (*p*–value <0.001) (26).

A significant difference between male and female knees has been published as well. Bellemens and coauthors found a mean FTMA 1.9° varus in males and 0.8° varus in females in a study (13). Deep and coauthors found the mean FTMA in males in extension was varus of 1.7°(SD = 4°), while in females it was varus of 0.4°(SD = 3.9°). This was significantly different from each other (*p*-value 0.010) (26).

Bellemans found 32% constitutional varus (more than 3°) in males and 17.2% in females (13). Deep found similar values for constitutional varus (more than 3°) in 33.7% in male and 20% in female knees (26).

We found that an interesting variation was seen between two races. Those from northern part of a country (Aryan Origin) had FTMA of valgus 0.41°(SD = 3.62°) and those from southern part of the country (Dravidian origin) had an FTMA of varus 1.28°(SD = 4.03°). These were found to be statistically significantly different (*p*-value 0.01).

An understanding of the normal knee alignment and its variations with weight-bearing and gender may help surgeons optimize outcomes after total knee arthroplasty in individual patients. However, the biomechanics of a replaced knee are different from a normal knee. In a replaced knee an attempt is made to achieve a neutral mechanical axis. This is aimed to achieve uniform loading of the medial and lateral compartments. Thus, the polyethylene insert on both the compartments should be equally stressed. This theoretically should lead to equal wear of the poly on both sides and avoid asymmetric wear that may lead to acceleration of wear and early failure. Recent use of computer navigation has been shown to be good at achieving this neutral alignment. However, implied results of the studies described above may indicate that neutral mechanical axis achieved in supine position during surgery of total knee arthroplasty is liable to change during weight bearing. This change has a tendency toward varus alignment and extension. This is supported by the other study showing that even in replaced knees this tendency to varus with standing is maintained (28). Hence, aiming for 1° or 2° of valgus may be appropriate to accommodate for the changes during weight bearing. However, this is only a suggestion and further studies are needed to make a good argument for this. From results of above studies, it is expected that, in sagittal plane, if you correct the straightening of leg up to 5° of flexion, at end of surgery, it may be acceptable as this tends to correct with weight-bearing standing posture. One needs to be careful in extrapolating results of normal knees to arthritic and replaced knees and further studies are required to confirm the clinical benefit.

## Collateral Ligament Laxity

We also quantified the collateral laxity in normal persons with a quantified stress test and compared gender differences (29).

It has been shown that non-weight-bearing alignment in lying position is different from weight-bearing standing position (7). This applies to both arthritic and replaced knees but more so to the former (27). Collateral soft tissue imbalance has been implicated as one of the factors affecting the weight-bearing alignment of the knee (27). The results of knee replacements are not only affected by the alignment but also the soft tissue balance. Instability after knee replacement is one of the major causes for failure. There is no consensus on the “normal” collateral ligament laxity. Traditionally surgeons are taught to have about 2° of collateral laxity in either direction at end of procedure, though there is no specific scientific basis of choosing this figure for every TKA. Some previous studies have reported that up to 2° of varus and valgus laxity is compatible with good results in PCL retaining TKA (30–32). The literature on living tissues normal knee collateral laxity is minimal (33). The understanding of normal knee alignment and mediolateral laxity in the knee joint specific to the individual patients is necessary to further improve outcomes after TKA. This will help surgeon to aim for a figure as to how tight the prosthetic knee should be balanced.

In a study on collateral laxity, on application of varus stress of 10 Newton Meters (NM) to the knee, the supine 0° flexion FTMA changed significantly by a mean varus of 3.1°(SD = 2°) (29). This was a statistically significant change (*p*-value <0.001). The supine FTMA at 15° flexion changed significantly by mean varus of 6.9°(SD = 2.6°) on varus stress, again a significant change (*p*-value <0.001) (29). On application of valgus stress of 10 Nm, the supine FTMA at 0° changed significantly by mean valgus of 4.6°(SD = 2.2°) (*p*-value <0.001) and at 15° flexion again changed significantly by valgus of 7.9°(SD = 3.4°) (*p*-value < 0.001) (29).

Heesterbeek et al. used a radiographic method and Telos tensioner with 15 NM torque and reported varus laxity of 2.8°(SD = 1.3) and valgus laxity of 2.3°(SD = 0.8) in full extension in normal patients (34). Yoo et al. have shown 6.7–7.2° varus laxity and 3.9–4.3° valgus laxity in 20° knee flexion in normal Korean patients using a custom-made measurement scale (Shinwa rules company ltd) (33). The results of all these studies are consistent in showing less collateral laxity in full extension as compared to 15° of flexion. This could be the effect of tissues, like posterior capsule, that are tight in extension but lax in flexion and, hence, the effect is negated in flexion measurements allowing more collateral laxity in flexion.

A significant variation in the laxity of the collateral ligaments has also been seen between males and females (29).

The question in TKA is what amount of laxity must be there to achieve a good results. Some studies have reported that 3–4° of varus and valgus laxity is compatible with good results in PCL retaining TKA (30–32). The effect of muscle tension on the values obtained must be considered because patients undergoing surgery are under anesthesia with lesser muscle tension and, therefore, more laxity on stress testing. Hence, it may be difficult to extrapolate the data obtained in these studies to the arthroplasty population but it can act as a rough guide. We found that the normal laxity in collaterals is much more than is conventionally used by surgeons. The wide variations in femorotibial mechanical axis and collateral laxity may in part account for the dissatisfaction in patients with mechanically aligned TKA. Achievement of less than normal laxity at the end of surgery may lead to tightening of tissues resulting in pain, discomfort, and stiffness. Knowledge of the normal FTMA and collateral ligament laxity can help the surgeon to achieve better balancing after total knee arthroplasty.

However, given the wide range of valgus and varus laxity, it may be useful to measure the healthier knee in patients with unilateral knee osteoarthritis. This measurement can then be reproduced at the time of surgery for replaced knee. One of the challenges during surgery is to assess the laxity and axis accurately. Instead of using spacers, surgeons apply tensioners and subjective feel, the intraoperative use of computer navigation system may enable the surgeon to reproduce the FTMA and collateral laxities more accurately as one will have numbers to work with, which can be seen real time during surgery.

## Discussion

Use of pre- and intraoperative computer navigation may help the surgeon set better goals of alignment and kinematics for individual patients and achieve desired results intraoperatively in those patients.

The wide variations in inherent FTMA and collateral laxity may in part account for the dissatisfaction in patients with mechanically neutral aligned TKR. Not only it changes with posture but it has been shown to change in majority of patients as the knee flexes from extended position (35). Knowledge of the normal dynamicity of FTMA can help the surgeon to achieve better alignment after total knee arthroplasty. The use of computer navigation measurement device can be a useful screening tool on outpatient basis, especially if other knee of the patient is normal. Also, the method can be used to evaluate and compare outcomes after total knee arthroplasty. However, the question still remains if we should aim for a constitutional alignment of a person (putting the polyethylene insert at risk of uneven medio-lateral loading) or we should aim for a neutral alignment that is not constitutionally normal for that person or we should aim for 1°–2° of valgus during surgery that will become neutral on standing. These questions need to be answered with further studies.

The small differences may not make huge clinical impact. However, if we strive to measure and try to reproduce this, it will avoid gross errors. Although it will lead to only small variations, it will make it better for the outliers, though only small clinical impact in the majority of cases. It is very hard to prove if these small differences will make a significant clinical impact.

In conclusion, the mean mechanical axis of knee in normal persons is not neutral and is in varus though varies in individual humans. The alignment of the knee is not static and is “dynamic” which changes with flexion and posture as one stands from a non-weight-bearing supine position. This also differs between individuals, gender, and race. The collateral ligament laxity is also variable in different humans and female ligaments are more lax than male ligaments. These findings may help surgeons go a long way in understanding of the normal human knees.

## Conflict of Interest Statement

The institution gets research funding from Stryker, Zimmer, BBraun Aesculap, Convatec, Blue Belt Technologies, unrelated to this article.
